# Case Report: Vortioxetine in the Treatment of Depressive Symptoms in Patients With Epilepsy—Case Series

**DOI:** 10.3389/fphar.2022.852042

**Published:** 2022-03-31

**Authors:** Marcin Siwek, Aleksandra Gorostowicz, Magdalena Bosak, Dominika Dudek

**Affiliations:** ^1^ Department of Affective Disorders, Jagiellonian University Medical College, Kraków, Poland; ^2^ Department of Psychiatry, Jagiellonian University Medical College, Kraków, Poland; ^3^ Department of Neurology, Jagiellonian University Medical College, Krakow, Poland

**Keywords:** vortioxetine, depression, bipolar disorder, seizure, antidepressants, epilepsy, mood disorders, depressive symptoms

## Abstract

Epilepsy and depression are both serious and potentially disabling conditions which often coexist—bidirectional relationship between the two disorders has been observed. Comorbidity between depression and epilepsy can be attributed to: underlying common pathophysiological mechanisms, psychiatric side effect of antiepileptic medications and psychological response to stress in people with chronic, neurological condition. Despite high prevalence of depressive symptoms in patients with epilepsy, current evidence of the effectiveness of antidepressant therapy in this group of patients is very limited. Vortioxetine is an antidepressant with multimodal activity, very good treatment tolerability, low risk of inducing pharmacokinetic interactions, relative safety of treatment in patients with somatic comorbidities, low risk of causing: sedation, sexual dysfunctions and metabolic side effects. Vortioxetine seems to be a promising treatment option for depressed patients with cognitive dysfunctions, anhedonia and anxiety. In this case series, we report nine cases of patients with epilepsy and depressive symptoms treated with vortioxetine. Seven cases are patients with secondary focal and generalized epilepsy and two with unclassified epilepsy. Three patients presented with depressive episode in the course of bipolar disorder and six patients had depressive symptoms due to organic mood disorder. The dose range of vortioxetine was between 10 and 20 mg. In all of the presented cases effectiveness and tolerability of treatment were very good. Remission of depressive symptoms was achieved in all patients. No epilepsy seizures after switch to vortioxetine were observed in seven cases. In two patients seizures occurred during the first months of vortioxetine treatment but this most probably was due to suboptimal antiepileptic treatment—satisfactory seizure control was achieved after optimization of antiepileptic pharmacotherapy. Vortioxetine was discontinued in two of the presented cases due to pregnancy planning. The duration of observation period during vortioxetine therapy ranged from 2 to 48 months. In conclusion, vortioxetine can be a promising treatment option in patients with epilepsy and comorbid depressive symptoms.

## Introduction

Epilepsy is a chronic, heterogenous neurological condition affecting more than 50 million people globally (Elger et al., 2017). It is defined by any of the following: (Elger et al., 2017) At least two unprovoked (or reflex) seizures occurring >24 h apart; (Fisher et al., 2014) one unprovoked (or reflex) seizure and at least 60% probability of further seizures, occurring over the next 10 years; (Josephson et al., 2017) diagnosis of an epilepsy syndrome (Fisher et al., 2014). Psychiatric disorders, especially depression, are considered to be prevalent comorbidities in patients with epilepsy. The risk of developing depression seems to be increased around twofold in epilepsy (compared to the general population) (Tellez-Zenteno et al., 2007; Josephson et al., 2017). Data considering the prevalence of depression in epilepsy patients is inconsistent, with results ranging from around 6% to more than 50% (Bosak et al., 2012; Bosak et al., 2015a; Błaszczyk and Czuczwar, 2016; Elger et al., 2017)—higher prevalence is observed in drug-refractory than in well-controlled epilepsy (Bosak et al., 2012; Garcia et al., 2015; Tao and Wang, 2016) The inter-ictal form is the most common manifestation of depression in epilepsy patients (Bosak et al., 2012; Błaszczyk and Czuczwar, 2016). Clinical presentation of mood disorders in this group of patients can be different from typical depressive episode—symptoms such as irritability, low frustration tolerance, mood lability, anxiety and fatigue are prevalent (Kwon and Park, 2014). Sometimes terms “interictal dysphoric disorder” or “dysthymic-like disorders of epilepsy” are used in the literature to describe mood disorders in epilepsy (Bosak et al., 2012; Tao and Wang, 2016).

Interestingly, bidirectional relationship between depression and epilepsy has been observed. Depression is associated with around 2.5 times increased hazard of epilepsy (Josephson et al., 2017), although some studies reported that the risk of developing epilepsy in patients with a history of depression can be even up to seven-fold higher (compared to the general population) (Kanner, 2009; Bosak et al., 2012; Błaszczyk and Czuczwar, 2016). Furthermore, more than two-fold increase in the incidence of treatment-resistant epilepsy has been observed in patients with preceding depression (Hitiris et al., 2007). Comorbid depression in epilepsy is also associated with reduced quality of life, poorer treatment adherence, premature mortality and higher risk of unemployment (Johnson et al., 2004; Taylor et al., 2011; Fazel et al., 2013; Garcia et al., 2015; Keezer et al., 2016; Henning et al., 2019). Lifetime history of psychiatric disorders may be a predictor of poor outcome after epilepsy surgery (Kanner et al., 2009). It is worth mentioning that people with epilepsy have even 5-fold increased risk of suicide (compared to controls) and this can be mediated by psychiatric disorders (Bosak et al., 2015b; Bosak et al., 2016; Hesdorffer et al., 2016).

Comorbidity between depression and epilepsy can be attributed to different underlying common pathophysiological mechanisms. Some of the suggested ones are: neuroinflammation, structural and functional brain abnormalities (in temporal and prefrontal areas), alterations in neurotransmission (particularly GABA, glutamate, serotonin, noradrenaline, dopamine transmission), hyperactivity in hypothalamic-pituitary-adrenal axis, shared genetic susceptibility (Bosak et al., 2012; Błaszczyk and Czuczwar, 2016; Mazarati and Sankar, 2016; Tao and Wang, 2016). Besides, a few antiseizure medications are known to potentially induce or exacerbate depressive symptoms, e.g., levetiracetam, topiramate, vigabatrin, barbiturates and zonisamide (Bosak et al., 2012; Błaszczyk and Czuczwar, 2016; Chen et al., 2017). Finally, depressive symptoms could potentially be an element of psychological response to stress, stigmatization and isolation due to living with epilepsy (Błaszczyk and Czuczwar, 2016).

Data considering efficacy and safety of antidepressants in treatment of depressive symptoms in epilepsy patients are relatively scarce—only a few randomized controlled trials have been published so far (Elger et al., 2017). According to the Cochrane Review from 2021, current evidence on the effectiveness of treatment with antidepressant medications in patients with epilepsy is very limited (Maguire et al., 2021). Relatively the largest amount of data refers to SSRIs (SSRI—Selective Serotonin Reuptake Inhibitors) which seem to be safe and effective (although heterogeneity of the results is significant) (Mula, 2019). Despite previous fears of a potential seizure-provoking action of antidepressants, data from more thorough analyses indicate that treatment with these medications is rather safe (with exceptions for bupropion, clomipramine and maprotiline) (Alper et al., 2007; Steinert and Fröscher, 2018; Mula, 2019). Moreover, a summary of FDA data from clinical trials showed that the incidence of seizures was lower in patients receiving antidepressants than placebo (standardized incidence ratio = 0.48) (Alper et al., 2007).

One of the latest therapeutic options for treatment of depressive symptoms is the multimodal antidepressant vortioxetine which has a very favorable safety and tolerance profile and a relatively low risk of drug interactions (Chen et al., 2018). Therefore, it seems reasonable to consider vortioxetine as a therapeutic option in patients with epilepsy and depressive symptoms.

Preclinical studies mostly indicate that vortioxetine may reduce seizure activity. Vortioxetine has been observed to reduce the number of epileptic discharges in penicillin-induced epilepsy model and to play a role in controlling epileptiform activity in pentylenetetrazole-induced kindling model in rats (Ögün et al., 2019; Taskiran and Unal, 2021). One study has shown proconvulsant activity of vortioxetine in genetic absence epileptic WAG/Rij rats (Aygun and Ayyildiz, 2021).

To the best of our knowledge, only one case of a patient with epilepsy (symptomatic occipital lobe epilepsy) successfully treated with vortioxetine has been published so far (Onder et al., 2018). In this case series, we report nine cases of patients with epilepsy and depressive symptoms treated with vortioxetine.

## Case Series—Results

All nine cases are outpatients treated in the Department of Psychiatry, University Hospital in Cracow (Poland) during the last 5 years. The cases were identified by thorough analysis of records from patient visits since 2015 until September 2021. Relevant socio-demographic and clinical data are presented in Table 1.

**TABLE 1:** Presentation of nine cases of patients with depressive symptoms and epilepsy treated with vortioxetine.

Patient	1	2	3	4	5	6	7	8	9
Gender	F	M	F	M	F	M	F	M	K
Age (at VOR initiation)	24	74	28	77	43	59	39	30	59
Neurological diagnosis (with ICD-10 code) Scheffer et al. (2017)	G40 Epilepsy	G40.2 Focal epilepsy	G40.2 Focal epilepsy	G40.2 Focal epilepsy	G40.2 Focal epilepsy	G40.2 Focal epilepsy	G40 Epilepsy	G40.2 Focal epilepsy	G40.2 Focal epilepsy
Epilepsy etiology	Unknown etiology	Structural (craniotomy for brain abscess in the temporal lobe)	Structural (surgery for oligoastrocytoma)	Structural (craniotomy for brain abscess in the temporal lobe)	Structural (posttraumatic)	Structural (poststroke)	Brain injury after cardiac arrest	Structural (surgery for astrocytoma in the temporal lobe)	Unknown etiology
Type of epilepsy seizures before VOR Tx (Fisher et al., 2017)	Tonic-clonic seizures	Focal seizures with impaired awareness	Focal seizures with and without impaired awareness, remission since the age of 20	Focal seizures with and without impaired awareness, 1 per 2 months	Focal seizures with impaired awareness, last one 2 years before. Just before VOR Tx the patient had focal to bilateral tonic-clonic seizure and status epilepticus due to medication overdose (suicidal attempt)	2 epilepsy seizures in the last months. Focal seizures with impaired awareness and focal to bilateral tonic-clonic seizure 1 per 3 months	Tonic-clonic seizures	Focal seizures with impaired awareness	Daily focal seizures with and without impaired awareness
DRE	No	No	No	Yes	No	Yes	No	Yes	Yes
Duration of previous antiseizure Tx (years)	1	5	12	2	14	1	4	15	28
ASM during VOR Tx	Levetiracetam 1,000 mg daily	Lamotrigine 400 mg, Levetiracetam 3 g daily	Lamotrigine 200 mg daily	Valproate 1,000 mg, Levetiracetam 500 mg daily	Lamotrigine (doses increased from 200 to 250 mg during treatment with VOR), Valproate (doses increased from 1,000 to 1,600 mg daily)	Valproate 2,000 mg daily, then switched to lamotrigine 200 mg daily	Lamotrigine 100 mg daily	Levetiracetam 1,500 mg daily	Valproate 1,000 mg daily, topiramate 62.5 mg daily
Mood disorder ICD-10 diagnosis	F31 Bipolar affective disorder	F31 Bipolar affective disorder	F31 Bipolar affective disorder	F06.3 Mood disorder due to known physiological condition	F06.3 Mood disorder due to known physiological condition	F06.3 Mood disorder due to known physiological condition	F06.3 Mood disorder due to known physiological condition	F06.3 Mood disorder due to known physiological condition	F06.3 Mood disorder due to known physiological condition
Total duration of psychiatric treatment	8 years	7 years	6 years	No psychiatric treatment before VOR initiation	13 years	4 years	9 years	2 months	8 years
Duration of the depressive episode before VOR initiation	3 months	3 weeks	3 weeks	6 months	3 weeks	1 month	1 month	1 month	1 year
Clinical picture of depression at VOR initiation	Fatigue, hypersomnia, cognitive dysfunctions, lowered mood	Lowered mood, apathy, anhedonia, generalized anxiety	Generalized anxiety, lowered mood, anhedonia, cognitive dysfunctions	Apathy, anhedonia, lowered mood, social withdrawal, deterioration of verbal fluency, irritability, fatigue	Anhedonia, generalized anxiety, cognitive dysfunctions, lowered mood, feeling of guilt, insomnia	Lowered mood, apathy, anhedonia, lack of energy, sexual dysfunctions	Lowered mood, apathy, anxiety	Anergy, apathy, anhedonia, amotivation, lowered mood, cognitive dysfunctions	Cognitive dysfunction, irritability, lowered mood, lack of energy, apathy, feeling of hopelessnes, anhedonia
Psychiatric comorbidity	—	F07 Personality and behavioural disorders due to brain disease, damage and dysfunction	—	—	F07 Personality and behavioural disorders due to brain disease, damage and dysfunction	—	F06.2 Psychotic disorder with delusions due to known physiological condition	F06.2 Psychotic disorder with delusions due to known physiological condition	—
		F10 Mental and behavioural disorders due to use of alcohol							—
		F13 Sedative, hypnotic, or anxiolytic related disorders					F04 Amnestic disorder due to known physiological condition		
Previously used antidepressants	Escitalopram	Duloxetine	—	—	Venlafaxine	Sertraline, escitalopram, duloxetine	—	—	citalopram
Reason for VOR initiation	Depression with cognitive dysfunctions. Insufficient effectiveness of escitalopram	Somatic comorbidities, depressive symptoms: anhedonia, anxiety. Lack of effectiveness of duloxetine	Depression with anhedonia, anxiety and cognitive dysfunctions	Depressive symptoms: anhedonia and cognitive dysfunctions	Depressive symptoms: anhedonia, anxiety and cognitive dysfunctions	Depressive symptoms: anhedonia, apathy, sexual dysfunctions and insufficient effectiveness of SSRI and SNRI medications	Depressive symptoms: apathy, anxiety, cognitive dysfunctions	Depressive symptoms: apathy, cognitive dysfunctions, anhedonia	Depressive symptoms: anhedonia, apathy, cognitive dysfunctions
					Insufficient effectiveness of venlafaxine				
VOR dosage (mg)	10	Initially 10 mg, increased to 20	10	10	15	10	10	10	10
Other simultaneously used psychiatric medications (daily doses)	Aripiprazole 10 mg	Naltrexone 50 mg 1 × 1 Trazodone XR 0-0-150 mg Sulpiride up to 350 mg Bromazepam up to 6 mg daily	—	—	Trazodone CR 0-0-150 mg	—	Haloperidol depot—50 mg i.m. every 3 weeks Biperiden 4 mg daily	Risperidone 1 mg Eszopiclone 1 mg	-
Somatic comorbidities	—	Non-insulin-dependent diabetes mellitus Hypertension	—	Ischemic heart disease	—	Chronic obstructive pulmonary disease, Migraine, nephrolithiasis, dyslipidemia	—	—	—
Other medications during VOR Tx	—	Amlodipine Lisinopril Hydro-chlorothiazide Eplerenone Nebivolol Dabigatran Levothyroxine Glimepiride	—	Amlodipine	—	Ciclesonide, formoterol, tiotropium, atorvastatine, acetylsalicylic acid	—	—	—

ASM, antiseizure medications, CR, controlled release, DRE, drug-resistant epilepsy [defined according to the consensus proposal of the ILAE Commission on Therapeutic Strategies as failure of adequate trials of two tolerated, appropriately chosen and used antiepileptic drug schedules (whether as monotherapies or in combination) to achieve sustained seizure freedom (Kwan et al., 2010)] F, female, ICD-10, International Classification of Diseases Version 10, i.m., intramuscular, M, male, SNRI, Serotonin Norepinephrine Reuptake Inhibitor, SSRI, Selective Serotonin Reuptake Inhibitor, Tx, treatment, VOR, vortioxetine, XR, extended release.

The presented group of cases includes five female and four male patients, aged between 24 and 77 year-old. Seven cases are patients with secondary focal and generalized epilepsy and two with unclassified epilepsy, with mostly satisfactory control of the neurological disease. In most cases (5/9) lamotrigine was used as part of the antiseizure pharmacotherapy, but four patients were treated with levetiracetam and one with topiramate—medications with, as already mentioned in the introduction, know potential to induce psychiatric side effects. However, due to good antiseizure effect, the attending neurologists decided to maintain treatment with the use of these medications. Before starting treatment with vortioxetine three patients presented with depressive episode in the course of bipolar disorder and six patients had depressive symptoms due to organic mood disorder (i.e., due to known physiological condition). Vortioxetine was the first line of antidepressant treatment in four patients, second line in four patients and the fourth line in one case. Vortioxetine was preferred either because of the clinical presentation (depression with anhedonia, anxiety and cognitive dysfunctions—in all patients), insufficient effectiveness of previously used antidepressants (in five patients) or somatic comorbidities and high risk of drug interactions due to polytherapy (in three patient). In the subgroup of patients with bipolar disorder, vortioxetine was chosen after analysis of the history of previous lack of antidepressant effectiveness of other agents (such as mood-stabilizers or antipsychotics) and taking into consideration relative safety of different pharmacological options when treating patients with epilepsy. Moreover, results of a naturalistic, open-label, add-on study of vortioxetine in bipolar depression indicate that this medication can be an efficient and well-tolerated therapeutic option in bipolar patients (Siwek et al., 2021a). It is also worth noticing that two out of three bipolar patients in our case series were treated with lamotrigine which was used not only due to its antiseizure but also antidepressant properties.

The dose range of vortioxetine was between 10 and 20 mg. In all presented cases remission of depressive symptoms was achieved and tolerability of the treatment was good. Time needed to reach remission ranged from 3 weeks to 3 months. No epilepsy seizures after introduction of vortioxetine were observed in seven cases. In two cases seizures occurred during the observation period. In one patient (Patient 5) epileptic seizure appeared after 7 months of the treatment and this could have been attributed to inadequately low doses of lamotrigine (which was previously used in doses up to 600 mg with optimal seizure control, but during the first months of vortioxetine treatment daily dose of lamotrigine was reduced by neurologists to only 200 mg)—after lamotrigine dose adjustments no seizures were observed for the following 5 months of observation. In another patient (Patient 6) epileptic seizure occurred after 2 months of vortioxetine treatment—antiseizure medication was switched afterwards (from valproate to lamotrigine) with optimal control: no other epileptic seizure was observed during nearly 4 following years of antidepressant treatment. It is worth noticing that possibly in these two cases no optimal control of epileptic seizures was reached prior to vortioxetine treatment—both patients had seizure episodes requiring hospitalization shortly before vortioxetine initiation. Vortioxetine was discontinued in two of the presented cases due to pregnancy planning, taking into consideration scarcity of data regarding safety of vortioxetine use in pregnant women. Duration of observation period ranged from 2 to 48 months.

Table 2 presents clinical data on the effectiveness and tolerability of treatment with vortioxetine.

**TABLE 2:** Effectiveness and tolerability of vortioxetine treatment in nine presented cases.

Patient	1	2	3	4	5	6	7	8	9
Psychiatric outcome of VOR Tx	remission	remission	remission	remission	remission	remission	remission	remission	Remission
Time to remission	3 months	2 months	2 months	2 months	3 weeks	2 months	1 month	2 months	2 months
Epilepsy course during VOR Tx	No epilepsy seizures	No epilepsy seizures	No epilepsy seizures	No epilepsy seizures	One epileptic seizure after 7 months of treatment (lamotrigine dose was increased consequently)	One epileptic seizure after approximately 2 months of treatment (valproate was afterwards switched to lamotrigine)	No epilepsy seizures	No epilepsy seizures	No epilepsy seizures
Duration of observation (months)	5 (VOR discontinued due to pregnancy planning)	38	6 (VOR discontinued due to pregnancy planning)	2	12	48	26	4	6
Treatment tolerability	good	good	good	good	good	Good	good	good	Good

Tx, treatment, VOR, vortioxetine.

## Discussion

In the presented cases vortioxetine was very well tolerated and effective (remission of depressive symptoms was reached in all patients), despite the fact that all patients were treated with polytherapy and three had somatic comorbidities (apart from epilepsy). It is worth mentioning that the majority of cases included patients with symptomatic epilepsy due to brain lesion (after stroke, cardiac arrest, brain injury or neurosurgery)—current data on effectiveness and tolerability of antidepressants in patients with depression following brain damage (due to, e.g., stroke or traumatic brain injury) is limited, which makes any reports of successful treatment of depressive episodes in this group of patients especially valuable (Qin et al., 2018; Kreitzer et al., 2019). Besides, two of the presented cases were elderly patients (74 and 77 year-old)—both reached remission in 2 months and tolerated vortioxetine very well, even though possible reduced tolerability and effectiveness of antidepressants in patients over the age of 65 has been observed (Cleare et al., 2015).

As already mentioned in the introduction, current evidence on the treatment of depression in epilepsy is limited and inconclusive. According to the available clinical recommendations, SSRI and SNRI (Serotonin Norepinephrine Reuptake Inhibitors) are the first-line pharmacological treatment options, while tricyclic antidepressants or NDRI (norepinephrine-dopamine reuptake inhibitors) should be avoided (Elger et al., 2017; Mula, 2019).

Vortioxetine is a relatively new antidepressant (approved by FDA—Food and Drug Administration, in 2013) with multimodal activity—it is an inhibitor of serotonin transporter, an agonist of 5HT1A receptor, a partial agonist of 5HT1B receptor, and an antagonist of 5HT1D, 5HT3 and 5HT7 receptors. Antidepressant efficacy has been demonstrated in doses 5–20 mg daily. It is metabolized primarily by CYP2D6 isoenzyme of P450 cytochrome (to inactive metabolites). Vortioxetine does not seem to impact activity of the liver enzymes in a clinically relevant way which potentially lowers the risk of pharmacokinetic drug interactions (Chen et al., 2018). Results of the network meta-analysis comparing efficacy and acceptability of 21 antidepressants in patients with MDD (Major Depressive Disorder) indicate that vortioxetine was in the group of more effective and more tolerable agents (Cipriani et al., 2018). Data from clinical trials has shown that vortioxetine carries relatively (compared to other antidepressants) low risk of inducing sexual dysfunctions, weight gain and sleep disturbance (Jacobsen et al., 2015; Jacobsen et al., 2016; D’Agostino et al., 2015; Liguori et al., 2019; Baldwin et al., 2016a). Besides, efficacy of vortioxetine on anhedonia and anxiety symptoms in patients with MDD has been observed (Baldwin et al., 2016b; Cao et al., 2019). Vortioxetine also appears to have relatively good tolerability and safety profile in depressed elderly patients (Borhannejad et al., 2020; Danielak, 2021).

Vortioxetine seems to be a promising treatment option for cognitive dysfunctions in MDD patients—it has been observed to improve cognition (both objectively and subjectively) independently of the alleviation of depression severity and to induce greater improvement in different cognitive domains compared to other antidepressants (Bennabi et al., 2019). Moreover, it has been demonstrated that vortioxetine added to a mood stabilizing agents can be an effective and well-tolerated treatment option in patients with bipolar depression (Siwek et al., 2021a). Finally, it is worth remembering that vortioxetine withdrawal can be associated with occurrence of discontinuation symptoms (although, contrary to the majority of SSRI and SNRI medications, the risk of withdrawal syndrome seems to be low) (Siwek et al., 2021b).

Seizures were very rarely observed in the published results of clinical trials of vortioxetine in MDD patients—only one randomized, double-blind 8-week trial reported 1 case of seizure occurrence in a patient receiving 5 mg of vortioxetine (out of 153 patients in this group and compared to no seizures observed in the placebo arm) (Mahableshwarkar et al., 2013).

Data considering use of vortioxetine in epilepsy patients is scarce. To the best of our knowledge, only one case of a 52-year-old female patient with secondary epilepsy and depressive symptoms has been presented—the authors reported excellent response to vortioxetine (in dose of 20 mg daily) and, interestingly, reduction of visual scotomas after initiation of antidepressant treatment (Onder et al., 2018). As patients with epilepsy often suffer from somatic and psychiatric comorbidities and thus may frequently require polytherapy, vortioxetine as a relatively safe, well-tolerated antidepressant with low risk of inducing pharmacokinetic interactions seems to be a valid treatment option in this group of patients (Baldwin et al., 2016a; Bosak et al., 2019). Besides, patients with epilepsy commonly suffer from cognitive impairment and it would be interesting to find out whether cognition enhancing properties of vortioxetine may additionally improve effects of treatment with this medication (Holmes, 2015).

## Conclusion

Vortioxetine, as an effective and relatively well-tolerated antidepressant with potentially lower risk of pharmacokinetic drug interactions and additional cognitive benefits in depression, seems to be a promising treatment option for patients with epilepsy and depressive symptoms (especially in the view of current limited evidence on the effectiveness of antidepressants in epilepsy patients). We have presented 9 cases of patients successfully treated with this medication—symptomatic remission was achieved with good treatment tolerance. Further studies regarding vortioxetine use in larger populations of patients with epilepsy and depression are definitely needed in order to broaden our knowledge and improve clinical management of this group of patients.

## Data Availability

The data analyzed in this study is subject to the following licenses/restrictions: The dataset contains clinical information about outpatients and cannot be openly available due to privacy restrictions. Requests to access these datasets should be directed to marcin.siwek@uj.edu.pl.
